# Supplementary material for: A Likelihood Approach for Real-Time Calibration of Stochastic Compartmental Epidemic Models
Source: PLoS Comput Biol. 2017 Jan 17;13(1):e1005257. doi: 10.1371/journal.pcbi.1005257 (PMC5240920; doi:10.1371/journal.pcbi.1005257)
Supplement: S1 File — (TAR.GZ) [file pcbi.1005257.s014.tar.gz › HSPH_Online-SI-Revision/PF08/n1-severe/n1-severe_PF2_table-8.pdf]

1	newly 3 weeks spec	19.	39.56	{3., 76.}	141.895	0.481451	newly 3 weeks cumul	34.	118.98	{16., 232.}	271.176	0.604227	newly 5 weeks spec	25.	35.09	{2., 66.}	91.96	0.49103	newly 5 weeks cumul	86.	191.77	{25., 356.}	154.5	0.515416	newly 62 weeks cumul	592.	424.7	{58., 752.}	41.8818	0.420914
2	newly 3 weeks spec	7.	46.81	{3., 103.}	589.857	0.791319	newly 3 weeks cumul	19.	133.65	{14., 282.}	616.895	0.751533	newly 5 weeks spec	33.	39.7	{3., 87.}	78.1818	0.475062	newly 5 weeks cumul	67.	217.84	{27., 435.}	249.284	0.538505	newly 64 weeks cumul	626.	458.58	{61., 803.}	39.1917	0.351253
3	newly 3 weeks spec	17.	45.37	{3., 92.}	202.412	0.585722	newly 3 weeks cumul	50.	131.17	{13., 265.}	196.46	0.514873	newly 5 weeks spec	23.	39.8	{3., 80.}	122.957	0.513559	newly 5 weeks cumul	88.	213.93	{25., 409.}	178.08	0.504866	newly 63 weeks cumul	618.	465.43	{60., 786.}	38.0372	0.344688
4	newly 3 weeks spec	5.	46.44	{2., 102.}	849.6	0.910453	newly 3 weeks cumul	13.	125.37	{8., 301.}	879.615	0.858287	newly 5 weeks spec	23.	49.56	{2., 92.}	154.696	0.550179	newly 5 weeks cumul	59.	223.58	{17., 480.}	304.644	0.620543	newly 66 weeks cumul	656.	567.94	{80., 842.}	33.3049	0.339034
5	newly 3 weeks spec	21.	44.3	{6., 97.}	150.	0.50587	newly 3 weeks cumul	47.	118.24	{13., 259.}	183.532	0.502947	newly 5 weeks spec	31.	44.84	{3., 88.}	97.4194	0.472931	newly 5 weeks cumul	110.	209.46	{26., 435.}	129.036	0.450214	newly 65 weeks cumul	613.	537.87	{102., 828.}	36.0179	0.303704
6	newly 3 weeks spec	13.	35.22	{1., 81.}	207.846	0.627916	newly 3 weeks cumul	22.	105.93	{5., 243.}	404.955	0.72311	newly 5 weeks spec	15.	32.56	{0., 66.}	164.133	0.64622	newly 5 weeks cumul	45.	172.67	{6., 356.}	311.444	0.684202	newly 62 weeks cumul	650.	394.1	{19., 748.}	47.0154	0.545574
7	newly 3 weeks spec	23.	38.67	{2., 81.}	113.957	0.517811	newly 3 weeks cumul	69.	113.81	{5., 247.}	110.565	0.491059	newly 5 weeks spec	34.	35.92	{1., 77.}	72.7647	0.555554	newly 5 weeks cumul	132.	187.24	{7., 358.}	92.0455	0.494345	newly 62 weeks cumul	490.	423.49	{37., 776.}	46.5449	0.444458
8	newly 3 weeks spec	11.	47.03	{2., 104.}	356.091	0.687104	newly 3 weeks cumul	28.	127.27	{8., 312.}	380.893	0.645177	newly 5 weeks spec	39.	49.99	{2., 92.}	79.7179	0.478126	newly 5 weeks cumul	95.	226.54	{17., 491.}	175.453	0.530169	newly 66 weeks cumul	644.	570.78	{80., 842.}	33.7702	0.3369
9	newly 3 weeks spec	13.	36.84	{1., 78.}	217.692	0.63059	newly 3 weeks cumul	27.	107.15	{5., 211.}	322.704	0.681135	newly 5 weeks spec	24.	35.79	{1., 80.}	106.875	0.572973	newly 5 weeks cumul	73.	179.28	{6., 349.}	179.918	0.587287	newly 62 weeks cumul	503.	428.64	{37., 795.}	46.7157	0.470795
10	newly 3 weeks spec	19.	38.8	{2., 78.}	142.105	0.534627	newly 3 weeks cumul	44.	111.8	{7., 219.}	184.364	0.558063	newly 5 weeks spec	47.	37.52	{2., 80.}	57.0638	0.523895	newly 5 weeks cumul	144.	187.67	{8., 349.}	77.4514	0.467382	newly 62 weeks cumul	637.	454.41	{55., 796.}	40.3721	0.446814
11	newly 3 weeks spec	13.	41.63	{14., 82.}	225.462	0.438283	newly 3 weeks cumul	42.	126.55	{45., 245.}	203.357	0.407125	newly 5 weeks spec	27.	36.3	{10., 69.}	68.	0.255992	newly 5 weeks cumul	89.	203.2	{66., 385.}	136.292	0.320149	newly 59 weeks cumul	567.	435.85	{163., 708.}	35.9277	0.221059
12	newly 3 weeks spec	17.	60.76	{1., 135.}	291.529	0.70444	newly 3 weeks cumul	30.	160.94	{4., 388.}	464.867	0.767778	newly 5 weeks spec	56.	55.4	{0., 102.}	61.4286	0.586094	newly 5 weeks cumul	124.	277.09	{4., 600.}	165.185	0.627284	newly 67 weeks cumul	637.	600.96	{4., 883.}	33.6609	0.441284
13	newly 3 weeks spec	11.	36.98	{1., 81.}	266.909	0.668182	newly 3 weeks cumul	31.	108.74	{5., 243.}	276.903	0.639431	newly 5 weeks spec	30.	34.91	{0., 77.}	79.7	0.575933	newly 5 weeks cumul	95.	179.53	{6., 356.}	128.705	0.535734	newly 62 weeks cumul	650.	408.45	{19., 776.}	45.8138	0.529804
14	newly 3 weeks spec	13.	60.13	{1., 128.}	393.462	0.766648	newly 3 weeks cumul	26.	159.06	{4., 388.}	539.615	0.802672	newly 5 weeks spec	24.	54.84	{0., 102.}	169.	0.676212	newly 5 weeks cumul	70.	273.59	{4., 596.}	322.471	0.737891	newly 67 weeks cumul	660.	586.87	{4., 876.}	32.6652	0.454408
15	newly 3 weeks spec	34.	36.01	{1., 68.}	68.9706	0.524514	newly 3 weeks cumul	78.	110.79	{5., 209.}	91.1923	0.456237	newly 5 weeks spec	40.	29.68	{0., 60.}	53.55	0.561524	newly 5 weeks cumul	154.	174.1	{10., 332.}	70.3506	0.43849	newly 61 weeks cumul	487.	380.9	{60., 661.}	40.2587	0.350287
16	newly 3 weeks spec	11.	60.79	{1., 137.}	481.545	0.805871	newly 3 weeks cumul	21.	159.36	{4., 400.}	683.143	0.854954	newly 5 weeks spec	31.	55.28	{0., 102.}	123.226	0.632114	newly 5 weeks cumul	74.	275.63	{4., 609.}	305.041	0.725847	newly 67 weeks cumul	620.	596.87	{4., 884.}	36.2823	0.453389
17	newly 3 weeks spec	34.	59.78	{1., 137.}	127.412	0.597369	newly 3 weeks cumul	57.	155.95	{4., 400.}	212.649	0.613745	newly 5 weeks spec	41.	54.8	{0., 106.}	86.6829	0.599591	newly 5 weeks cumul	126.	269.79	{4., 609.}	156.865	0.616225	newly 67 weeks cumul	668.	587.02	{4., 884.}	34.1257	0.458855
18	newly 3 weeks spec	10.	59.23	{1., 128.}	519.5	0.822527	newly 3 weeks cumul	22.	157.05	{4., 388.}	638.318	0.835677	newly 5 weeks spec	22.	53.64	{0., 102.}	182.909	0.683846	newly 5 weeks cumul	59.	269.35	{4., 596.}	385.915	0.772099	newly 67 weeks cumul	630.	592.43	{4., 874.}	34.081	0.44335
19	newly 3 weeks spec	31.	48.1	{15., 92.}	78.8387	0.249877	newly 3 weeks cumul	69.	141.26	{49., 249.}	118.232	0.302601	newly 5 weeks spec	48.	41.61	{12., 77.}	43.2292	0.25719	newly 5 weeks cumul	148.	230.07	{72., 409.}	76.1689	0.240678	newly 60 weeks cumul	633.	482.57	{209., 772.}	33.1201	0.199688
20	newly 3 weeks spec	33.	48.74	{12., 94.}	81.2727	0.284123	newly 3 weeks cumul	87.	144.75	{46., 254.}	92.1034	0.2845	newly 5 weeks spec	54.	42.87	{14., 83.}	47.0926	0.298188	newly 5 weeks cumul	182.	234.7	{80., 431.}	63.7143	0.246634	newly 60 weeks cumul	622.	498.27	{219., 791.}	34.4807	0.200817
21	newly 3 weeks spec	27.	57.53	{1., 137.}	161.222	0.615759	newly 3 weeks cumul	47.	153.93	{4., 406.}	264.277	0.641716	newly 5 weeks spec	26.	51.4	{0., 102.}	144.154	0.649994	newly 5 weeks cumul	97.	261.28	{4., 627.}	206.907	0.64343	newly 67 weeks cumul	609.	575.12	{4., 884.}	39.4975	0.471351
22	newly 3 weeks spec	20.	31.84	{2., 67.}	109.1	0.50647	newly 3 weeks cumul	51.	98.08	{6., 202.}	135.059	0.478242	newly 5 weeks spec	65.	26.14	{3., 55.}	62.1846	0.688808	newly 5 weeks cumul	155.	153.07	{16., 297.}	63.271	0.420786	newly 61 weeks cumul	627.	357.57	{58., 661.}	47.1467	0.443032
23	newly 3 weeks spec	22.	44.49	{3., 87.}	142.409	0.504787	newly 3 weeks cumul	61.	138.25	{13., 263.}	163.23	0.487774	newly 5 weeks spec	31.	38.1	{3., 76.}	77.2903	0.480336	newly 5 weeks cumul	113.	219.32	{25., 410.}	133.646	0.467905	newly 63 weeks cumul	543.	446.49	{58., 761.}	38.5028	0.334633
24	newly 3 weeks spec	19.	30.9	{1., 66.}	109.158	0.524134	newly 3 weeks cumul	60.	105.06	{5., 194.}	115.6	0.461397	newly 5 weeks spec	37.	24.98	{1., 56.}	56.3243	0.570121	newly 5 weeks cumul	115.	158.68	{9., 292.}	85.7391	0.432254	newly 60 weeks cumul	485.	317.11	{38., 586.}	46.1464	0.43836
25	newly 3 weeks spec	31.	36.51	{10., 73.}	61.3226	0.312013	newly 3 weeks cumul	67.	110.45	{37., 229.}	86.6119	0.260525	newly 5 weeks spec	70.	31.14	{10., 65.}	58.7714	0.56733	newly 5 weeks cumul	176.	175.54	{62., 358.}	51.7727	0.260481	newly 58 weeks cumul	653.	396.93	{138., 682.}	43.3553	0.344811
26	newly 3 weeks spec	28.	43.99	{6., 97.}	106.321	0.477075	newly 3 weeks cumul	57.	117.43	{13., 259.}	142.93	0.466473	newly 5 weeks spec	43.	44.36	{3., 88.}	68.3721	0.464676	newly 5 weeks cumul	130.	207.6	{26., 435.}	104.2	0.429833	newly 65 weeks cumul	587.	534.04	{102., 828.}	37.8773	0.30548
27	newly 3 weeks spec	19.	50.75	{3., 104.}	198.895	0.530274	newly 3 weeks cumul	36.	145.84	{14., 290.}	326.556	0.604318	newly 5 weeks spec	37.	42.89	{2., 90.}	71.8649	0.453718	newly 5 weeks cumul	94.	236.24	{24., 453.}	180.574	0.496253	newly 64 weeks cumul	546.	466.81	{80., 803.}	40.6172	0.336084
28	newly 3 weeks spec	3.	60.3	{1., 135.}	1930.	1.2018	newly 3 weeks cumul	9.	159.83	{4., 388.}	1692.78	1.11975	newly 5 weeks spec	27.	54.93	{0., 102.}	144.333	0.647415	newly 5 weeks cumul	65.	274.84	{4., 600.}	353.015	0.755681	newly 67 weeks cumul	649.	599.92	{4., 876.}	32.5177	0.439167
29	newly 3 weeks spec	26.	44.59	{16., 82.}	90.8077	0.274686	newly 3 weeks cumul	77.	139.99	{46., 254.}	98.6364	0.279401	newly 5 weeks spec	43.	37.61	{15., 71.}	44.7674	0.27513	newly 5 weeks cumul	154.	218.	{77., 380.}	66.2208	0.229685	newly 60 weeks cumul	551.	461.02	{237., 740.}	30.824	0.175055
30	newly 3 weeks spec	38.	35.61	{1., 68.}	59.7105	0.519917	newly 3 weeks cumul	100.	108.89	{5., 209.}	66.93	0.446684	newly 5 weeks spec	52.	29.12	{1., 57.}	51.8846	0.569763	newly 5 weeks cumul	190.	171.	{10., 314.}	54.7789	0.428663	newly 61 weeks cumul	601.	379.18	{70., 639.}	41.9168	0.384593
31	newly 3 weeks spec	14.	38.3	{3., 77.}	205.143	0.575609	newly 3 weeks cumul	56.	113.63	{7., 229.}	139.018	0.50562	newly 5 weeks spec	13.	36.16	{2., 75.}	211.231	0.597912	newly 5 weeks cumul	98.	186.85	{8., 349.}	128.541	0.512237	newly 62 weeks cumul	505.	426.62	{37., 769.}	45.3426	0.440646
32	newly 3 weeks spec	13.	48.39	{3., 104.}	299.	0.643891	newly 3 weeks cumul	24.	139.46	{14., 278.}	496.833	0.701612	newly 5 weeks spec	8.	41.25	{4., 87.}	438.375	0.70941	newly 5 weeks cumul	57.	226.07	{27., 435.}	316.579	0.591281	newly 64 weeks cumul	509.	468.53	{61., 794.}	41.2515	0.307959
33	newly 3 weeks spec	19.	61.	{1., 137.}	258.632	0.684884	newly 3 weeks cumul	30.	159.33	{4., 400.}	460.3	0.762382	newly 5 weeks spec	36.	55.4	{0., 106.}	102.667	0.614887	newly 5 weeks cumul	97.	275.72	{4., 609.}	221.134	0.667628	newly 67 weeks cumul	689.	597.85	{4., 884.}	31.3687	0.447354
34	newly 3 weeks spec	23.	25.51	{2., 60.}	70.0435	0.423752	newly 3 weeks cumul	76.	83.69	{10., 167.}	69.6711	0.378645	newly 5 weeks spec	29.	19.3	{0., 44.}	59.3793	0.588087	newly 5 weeks cumul	132.	126.35	{12., 261.}	59.7652	0.371727	newly 59 weeks cumul	459.	287.42	{33., 569.}	50.8322	0.449423
35	newly 3 weeks spec	15.	59.32	{1., 137.}	328.267	0.720229	newly 3 weeks cumul	32.	155.5	{4., 400.}	414.688	0.734676	newly 5 weeks spec	45.	54.21	{0., 106.}	78.5111	0.602678	newly 5 weeks cumul	99.	268.17	{4., 609.}	207.081	0.648431	newly 67 weeks cumul	642.	583.47	{4., 884.}	36.5218	0.465443
36	newly 3 weeks spec	67.	62.16	{4., 151.}	73.9701	0.570914	newly 3 weeks cumul	106.	166.67	{11., 426.}	120.16	0.523422	newly 5 weeks spec	61.	56.16	{2., 119.}	65.2459	0.544872	newly 5 weeks cumul	222.	280.68	{25., 621.}	87.0811	0.501659	newly 68 weeks cumul	649.	613.15	{176., 861.}	30.2111	0.32038
37	newly 3 weeks spec	27.	42.87	{13., 88.}	80.6296	0.259623	newly 3 weeks cumul	77.	131.35	{50., 236.}	86.0909	0.251917	newly 5 weeks spec	49.	35.58	{9., 67.}	45.8367	0.319617	newly 5 weeks cumul	165.	205.22	{77., 377.}	53.3455	0.207851	newly 58 weeks cumul	598.	428.16	{148., 723.}	37.8395	0.254664
38	newly 3 weeks spec	10.	48.04	{2., 104.}	406.4	0.720501	newly 3 weeks cumul	38.	132.3	{8., 302.}	279.368	0.592818	newly 5 weeks spec	51.	49.72	{2., 92.}	60.4314	0.480463	newly 5 weeks cumul	123.	231.21	{17., 480.}	129.634	0.4933	newly 66 weeks cumul	597.	562.	{80., 842.}	37.5477	0.343751
39	newly 3 weeks spec	24.	37.74	{2., 81.}	106.333	0.531294	newly 3 weeks cumul	41.	111.13	{5., 243.}	203.293	0.583982	newly 5 weeks spec	32.	35.38	{0., 77.}	77.375	0.57728	newly 5 weeks cumul	105.	183.17	{6., 356.}	119.305	0.539853	newly 62 weeks cumul	520.	417.71	{19., 776.}	45.4712	0.484383
40	newly 3 weeks spec	29.	62.28	{4., 151.}	160.897	0.554145	newly 3 weeks cumul	61.	166.97	{11., 426.}	215.23	0.546765	newly 5 weeks spec	48.	56.71	{2., 119.}	78.1042	0.522994	newly 5 weeks cumul	145.	281.9	{25., 621.}	137.793	0.516456	newly 68 weeks cumul	580.	614.4	{176., 861.}	36.4966	0.331437
41	newly 3 weeks spec	22.	42.61	{2., 97.}	137.318	0.517488	newly 3 weeks cumul	38.	115.35	{12., 259.}	233.5	0.563788	newly 5 weeks spec	81.	42.79	{3., 88.}	52.8025	0.577155	newly 5 weeks cumul	156.	201.39	{18., 424.}	82.9167	0.446862	newly 65 weeks cumul	649.	531.48	{84., 828.}	34.5116	0.337715
42	newly 3 weeks spec	18.	46.02	{5., 97.}	190.222	0.547397	newly 3 weeks cumul	47.	127.95	{13., 271.}	204.489	0.536973	newly 5 weeks spec	26.	43.92	{3., 88.}	113.615	0.502278	newly 5 weeks cumul	102.	217.27	{18., 431.}	149.775	0.497492	newly 65 weeks cumul	610.	531.99	{83., 825.}	33.4738	0.326757
43	newly 3 weeks spec	35.	37.45	{11., 73.}	57.1143	0.286641	newly 3 weeks cumul	83.	113.6	{38., 232.}	67.012	0.236946	newly 5 weeks spec	42.	31.3	{5., 61.}	51.1905	0.438959	newly 5 weeks cumul	155.	178.93	{58., 365.}	56.4323	0.242057	newly 58 weeks cumul	514.	393.03	{63., 648.}	39.8541	0.287807
44	newly 3 weeks spec	15.	45.56	{2., 102.}	236.667	0.602508	newly 3 weeks cumul	34.	123.69	{8., 301.}	294.029	0.593494	newly 5 weeks spec	30.	48.6	{2., 92.}	107.2	0.501325	newly 5 weeks cumul	77.	220.15	{17., 480.}	217.571	0.557892	newly 66 weeks cumul	567.	561.3	{80., 842.}	40.2575	0.346892
45	newly 3 weeks spec	32.	46.15	{3., 90.}	94.0938	0.479604	newly 3 weeks cumul	68.	141.57	{18., 277.}	144.368	0.455387	newly 5 weeks spec	56.	37.54	{3., 78.}	51.3571	0.522072	newly 5 weeks cumul	159.	222.11	{31., 412.}	85.7673	0.402474	newly 63 weeks cumul	632.	445.98	{77., 758.}	37.9684	0.344171
46	newly 3 weeks spec	21.	36.84	{2., 81.}	118.19	0.524374	newly 3 weeks cumul	37.	107.98	{5., 243.}	219.892	0.589528	newly 5 weeks spec	19.	34.75	{0., 77.}	129.842	0.575036	newly 5 weeks cumul	68.	178.41	{6., 356.}	193.279	0.587099	newly 62 weeks cumul	566.	407.78	{37., 776.}	44.7173	0.490374
47	newly 3 weeks spec	13.	62.16	{4., 151.}	405.692	0.694905	newly 3 weeks cumul	21.	166.67	{11., 426.}	713.	0.77592	newly 5 weeks spec	26.	56.16	{2., 119.}	155.385	0.566613	newly 5 weeks cumul	64.	280.68	{25., 621.}	364.313	0.670505	newly 68 weeks cumul	615.	613.15	{176., 861.}	32.9545	0.324921
48	newly 3 weeks spec	37.	40.22	{3., 76.}	59.3514	0.419327	newly 3 weeks cumul	81.	121.81	{23., 243.}	90.5309	0.440719	newly 5 weeks spec	34.	36.59	{2., 66.}	67.4412	0.469996	newly 5 weeks cumul	153.	197.22	{26., 379.}	73.4771	0.437356	newly 62 weeks cumul	483.	433.73	{58., 769.}	44.3333	0.416748
49	newly 3 weeks spec	13.	47.11	{2., 104.}	293.	0.647149	newly 3 weeks cumul	42.	128.05	{8., 312.}	239.976	0.565196	newly 5 weeks spec	46.	49.96	{2., 92.}	66.6087	0.473118	newly 5 weeks cumul	113.	227.27	{17., 491.}	142.487	0.506015	newly 66 weeks cumul	632.	567.08	{80., 842.}	34.5285	0.338002
50	newly 3 weeks spec	9.	46.89	{3., 99.}	442.778	0.72747	newly 3 weeks cumul	25.	134.22	{14., 277.}	453.68	0.672241	newly 5 weeks spec	19.	40.62	{4., 87.}	154.211	0.516479	newly 5 weeks cumul	58.	219.47	{27., 433.}	298.603	0.572565	newly 64 weeks cumul	488.	468.26	{61., 803.}	43.4098	0.309528
